# Importance of Blood Cultures in Pneumonia

**Published:** 1919

**Authors:** 


***Importance of Blood Cultures in Pneumonia.*** By J. E. McClelland, Captain M. C., U. S. Army. *The Journal of the American Medical Association*, October 19, 1918.

The author records the observations made at the base hospital, Camp Beauregard, Alexandria, La., and draws the following conclusions:

Blood cultures in pneumonia are valuable from the standpoint of prognosis, and as a guide in the serum therapy of the Type 1 cases.

Septicemia is more common with the more virulent strains of pneumococcus and the streptococcus hemolyticus.

Type 1 pneumococcus septicemia responds very promptly to immune serum treatment. One may recover quickly from a moderately severe Type 4 pneumococcus septicemia.

The mortality in hemolytic streptococcus septicemia is very high, but one case has been reported in which the patient had a slight transient septicemia and recovered.

